# Microcapsules of Shrimp Oil Using Kidney Bean Protein Isolate and κ-Carrageenan as Wall Materials with the Aid of Ultrasonication or High-Pressure Microfluidization: Characteristics and Oxidative Stability

**DOI:** 10.3390/foods11101431

**Published:** 2022-05-16

**Authors:** Saqib Gulzar, Amjad K. Balange, Ravishankar Chandragiri Nagarajarao, Qiancheng Zhao, Soottawat Benjakul

**Affiliations:** 1 International Center of Excellence in Seafood Science and Innovation, Faculty of Agro-Industry, Prince of Songkla University, Songkhla 90110, Thailand; sgwani@hotmail.com; 2 QC Laboratory, Post-Harvest Technology, ICAR-Central Institute of Fisheries Education, Mumbai 400061, India; amjadbalange@cife.edu.in; 3 ICAR-Central Institute of Fisheries Education, Andheri West, Mumbai 400061, India; cnrs2000@gmail.com; 4 College of Food Science and Engineering, Dalian Ocean University, Dalian 116023, China; qczhao@dlou.edu.cn

**Keywords:** shrimp oil, kidney bean protein, κ-carrageenan, encapsulation, oxidation

## Abstract

Emulsions containing shrimp oil (SO) at varying amounts were prepared in the presence of red kidney bean protein isolate (KBPI) and κ-carrageenan (KC) at a ratio of 1:0.1 (*w*/*w*). The emulsions were subjected to ultrasonication and high-pressure microfluidization to assist the encapsulation process. For each sample, ultrasonication was carried out for 15 min in continuous mode at 80% amplitude, whereas high-pressure microfluidization was operated at 7000 psi for 10 min. Ultrasonicated and microfluidized emulsions were finally spray-dried to prepare KBPI-KC-SO microcapsules. Moderate to high encapsulation efficiency (EE) ranging from 43.99 to 89.25% of SO in KPBI-KC-SO microcapsules was obtained and the microcapsules had good flowability. Particle size, PDI and zeta potential of KBPI-KC-SO microcapsules were 2.58–6.41 µm, 0.32–0.40 and −35.95–−58.77 mV, respectively. Scanning electron microscopic (SEM) images visually demonstrated that the wall material/SO ratio and the emulsification method (ultrasonication vs microfluidization) had an impact on the size, shape and surface of the KBPI-KC-SO microcapsules. Encapsulation of SO in microcapsules was validated empirically using Fourier transform infrared (FTIR) analysis. Encapsulation of SO in KBPI-KC microcapsules imparted superior protection against oxidative deterioration of SO as witnessed by the higher retention of polyunsaturated fatty acids (PUFAs) and astaxanthin when compared to unencapsulated SO during extended storage at room temperature.

## 1. Introduction

Marine oils have long been known for their health benefits and nutraceutical properties. Oil derived from shrimp cephalothorax is a potential source of bioactive compounds, including polyunsaturated fatty acids (PUFAs), especially eicosapentaenoic acid (EPA) and docosahexaenoic acid (DHA), and an enormously potent antioxidant, namely astaxanthin. PUFAs are well documented for their anti-inflammatory and anti-tumor effects, and can help in the prevention of cardiovascular diseases [1,2]. The health benefits of astaxanthin include anti-cancer and anti-tumor effects, mainly due to its exceptional antioxidant activity [1]. Miki [3] reported that the antioxidant activity of astaxanthin was 100-fold greater than that of α-tocopherol. The presence of these valuable bioactive compounds has aroused a lot of research interest on the extraction and stabilization of shrimp oil. However, the fortification of shrimp oil in foods has been limited due to its high susceptibility to oxidation, resulting in the formation of a rancid odor and an off-flavor [4].

Over the years, encapsulation of oils and bioactive compounds using natural occurring polymers has been proven to be an effective technique for preventing their deterioration. Marine oils have been widely encapsulated in biopolymers using several methods, including spray-drying microencapsulation, liposomal encapsulation, complex-coacervation, nanoemulsions, etc. [4,5,6,7,8]. Spray-drying microencapsulation has been proven to be an effective delivery system for encapsulating oils within microcapsules for several reasons, including low operational cost, high throughput, continuous process and high loading capacity [9]. The efficiency of loading the oil into the core of microcapsules depends on numerous factors involving emulsion stability, size of emulsion droplets, type of wall material, ratio of wall material to oil and spray-drying conditions [10]. 

The capability of forming fine emulsions is a key factor for enhanced encapsulation efficiency [11]. Ultrasonication and high-pressure microfluidization have been implemented for the preparation of fine emulsions to enhance the encapsulation of bioactive compounds [12,13,14]. The use of high intensity ultrasound resulted in homogenous and smaller droplets, thus enhancing the stability of emulsions [15]. Ultrasound waves induce physical disruption of emulsion droplets by the acoustic cavitation effect, thereby reducing the droplet size [16]. Spray-drying microencapsulation of annatto seeds produced a high encapsulation efficiency when emulsions were subjected to sonication prior to spray-drying [13]. Another widely used technique for emulsification is high-pressure microfluidization, which channelizes the emulsion through micro-channels to produce nano-sized emulsions [17]. Many studies have documented that the emulsions prepared by microfluidization had superior droplet size distribution compared to conventional homogenization [18]. Verma et al. [19] reported that the encapsulation efficiency of microfluidized curcumin emulsion was significantly higher than that of its homogenized counterpart. Emulsions produced by two different methods including ultrasonication and microfluidization had different effects on the microencapsulation efficiency [20]. Microfluidization prior to spray-drying resulted in minimum unencapsulated oil at the surface of the microcapsules, whereas ultrasonication created nano-emulsions with high emulsion stability [21]. 

The utilization of proteins and carbohydrates for encapsulation of oil is not uncommon. Protein-polysaccharides have been extensively studied as wall materials for spray-drying microencapsulation [22]. Due to the amphiphilic nature of proteins, they form stable oil-in-water emulsions, and the subsequent drying of these emulsions result in the formation of dense protein network structures around the oil. Further combination of a polysaccharide with the protein in the wall materials has shown synergistic effects in terms of encapsulating efficiency [23]. Protein-polysaccharide complexes have high surface activity and can stabilize emulsions by increasing viscosity [24]. Plant-based proteins are in huge demand nowadays due to superior health benefits and sustainability compared to animal proteins. Due to the widespread acceptability, low-allergenicity and low-cost of plant-based proteins, especially legume proteins, they have been extensively used in the food industry [25,26]. Kidney bean protein isolate showed better gelation and emulsifying ability than other bean isolates [27]. κ-carrageenan, an anionic sulfated polysaccharide is widely used as a thickening and stabilizing agent. κ-carrageenan and kidney bean protein isolate can be used synergistically to stabilize an emulsion containing shrimp oil prior to spray drying to prepare microcapsules.

To our knowledge, there is no information on the spray-drying microencapsulation of shrimp oil (SO) using kidney bean protein isolate (KBPI) and κ-carrageenan (KC) as wall materials. Therefore, the aim of this study was to prepare emulsions of KBPI-KC-SO by ultrasonication and microfluidization and subsequently spray-dry to encapsulate SO using KBPI-KC as the wall material. The resultant microcapsules loaded with SO were characterized and studied for oxidative stability during extended storage.

## 2. Materials and Methods

### 2.1. Materials

Shrimp oil (SO) was extracted from cephalothorax of Pacific white shrimp following the method of Gulzar and Benjakul [28]. Red kidney beans were purchased from a local market. κ-carrageenan (KC) was procured from Krungthepchemi Co., Ltd., Lat Phrao, Bangkok, Thailand. Potassium chloride, potassium iodide, and sodium thiosulfate were obtained from Merck (Darmstadt, Germany). Acetic acid, methanol, ethanol, chloroform, n-hexane, hydrochloric acid and sodium hydroxide were purchased from Lab-Scan (Bangkok, Thailand). Gas chromatography standards were procured from Agilent Technologies (Santa Clara, CA, USA). Astaxanthin standard was obtained from Sigma-Aldrich (St. Louis, MO, USA).

### 2.2. Preparation of Kidney Bean Protein Isolate

Kidney bean protein isolate (KBPI) was prepared following the method of Fan and Sosulski [29]. Dehulled kidney beans were ground and subsequently sieved to obtain a fine flour, followed by lipid removal with 10 portions of butyl alcohol for 1 h. The defatted kidney bean flour was then dispersed in 20 parts of distilled water (DW), adjusted to pH 9 and stirred at room temperature for 1 h. After being centrifuged (10,000× *g* for 15 min at 4 °C), the supernatant was adjusted to pH of 4.5 and centrifuged at 10,000× *g* for 15 min at 4 °C. The precipitate was washed with 10 volumes of DW and centrifuged at 10,000× *g* for 15 min. The pellet was then lyophilized and named KBPI.

### 2.3. Preparation of KBPI-KC-SO Emulsions

One gram (1 g) of KBPI was dispersed in 100 mL of DW and the pH was adjusted to 10 to solubilize KBPI followed by the addition of 0.1 g KC to the solution. Finally, SO was added to the above solution at 0.1, 0.5 and 1% (*v*/*v*) under vigorous stirring at 30 °C for 1 h to obtain a homogenous mixture.

### 2.4. Ultrasonication and High-Pressure Microfluidization of Emulsions

Each KBPI-KC-SO mixture was subjected to ultrasonication and high-pressure microfluidization. Ultrasonication was carried out for 15 min in continuous mode at 80% amplitude using an Ultrasonic Processor (Vibra-CellTM VC 750, Sonics & Materials Inc., Newtown, CT, USA). Microfluidization was performed by forcing the emulsions through a high-pressure microfluidizer (Microfluidics, Model HC 5000, Stanwood, WA, USA) at 7000 psi for 10 min.

### 2.5. Spray-Drying Microencapsulation of Ultrasonicated and Microfluidized KBPI-KC-SO Emulsions

The ultrasonicated and microfluidized KBPI-KC-SO emulsions were spray-dried using a laboratory scale spray-dryer (LabPlant Ltd., LabPlant SD-05, Huddersfield, UK). The samples were fed to the drying chamber by a peristaltic pump at the feed rate of 5 mL min^−1^. The inlet temperature was kept at 180 ± 2 °C with the air flow rate of 4.3 m s^−1^ and the outlet temperature was 105 ± 2 °C. The resultant powders or microcapsules obtained from the spray-drying of ultrasonicated and microfluidized KBPI-KC-SO emulsions containing 0.1, 0.5 and 1% SO were labelled as US1, US2, US3, MF1, MF2 and MF3, respectively.

### 2.6. Characterization of Microcapsules

#### 2.6.1. Particle Size, Poly-Dispersity Index (PDI) and Zeta Potential

A PALS zeta potential analyzer (Brookhaven instruments corp, Holtsville, NY, USA) was used to determine the particle size, PDI and zeta (ζ) potential of KBPI-KC-SO microcapsules. Microcapsule samples were suitably diluted in ethanol and measured at 25 °C for size, PDI and ζ potential.

#### 2.6.2. Encapsulation Efficiency (EE)

The amount of SO encapsulated inside the KBPI-KC-SO microcapsules was measured following the procedure of Gulzar et al. [30]. EE was measured by quantifying surface oil and total oil for the microcapsules. EE was then calculated as follows:

$$\mathrm{EE}=\frac{\mathrm{To}-\mathrm{So}}{\mathrm{To}}\times 100$$

where To and So are total oil content and surface oil content, respectively.

#### 2.6.3. Scanning Electron Microscopic (SEM) Images

Microstructures of KBPI-KC-SO microcapsules were analyzed using a SEM (Quanta 400, FEI, Eindhoven, the Netherlands). An acceleration voltage of 15 kV and 5–10 Pa pressure were used for visualization of samples with a magnification of 5000×.

#### 2.6.4. Flowability

Flowability of KBPI-KC-SO microcapsules was determined empirically using the Hausner ratio (HR), which was calculated as follows:

$$\mathrm{HR}=\frac{\rho t}{\rho l}$$

where ρt is the tapped bulk density of the microcapsule samples and ρl is the loose bulk density of microcapsules.

#### 2.6.5. Fourier Transform Infrared (FTIR) Spectra

KBPI-KC-SO microcapsules that yielded the desirable encapsulation efficiency and flowability were selected for FTIR analysis. In addition, KBPI, KC and SO were also analyzed using a FTIR spectrometer (Bruker Model Equinox 55, Bruker Co., Ettlingen, Germany). Spectra in the range of 4000–400 cm^−1^ (mid-infrared region) were collected by 32 scans at a resolution of 4 cm^−1^.

### 2.7. Oxidative Stability of SO Encapsulated in KBPI-KC Microcapsules

SO and selected KBPI-KC-SO microcapsules were placed in zip-lock bag and stored at room temperature (28–30 °C) for 30 days. SO and oil extracted from KBPI-KC microcapsules, as described previously, were analyzed for lipid oxidation at day 0 and 30.

#### 2.7.1. Lipid Oxidation

The peroxide value (PV) of oil samples was measured by the titration method as detailed by Pudtikajorn and Benjakul [31]. Thiobarbituric acid reactive substances (TBARS) were determined following the method of Gulzar and Benjakul [32].

#### 2.7.2. Fatty Acid Profile

Fatty acid profiles expressed as fatty acid methyl esters (FAMEs) were determined using gas chromatography (GC) (Agilent GC 7890B; Santa Clara, CA, USA) fitted with a flame ionization detector (FID) as detailed by Gulzar and Benjakul [33]. The lipid samples (0.1 g) were mixed with 200 μL of 2 M methanolic sodium hydroxide and vortexed. The prepared solution was heated at 50 °C and 200 μL of 2 M methanolic hydrochloric acid were added to obtain fatty acid methyl esters (FAME). The FAMEs were injected into the GC at an injection temperature of 250 °C. Fatty acid content was calculated on the basis of the peak area ratio and expressed as the percentage.

#### 2.7.3. Astaxanthin 

Astaxanthin content in shrimp oil samples was analyzed following the method of Gulzar and Benjakul [28]. High-performance liquid chromatography (Waters 2475, Milford, MA, USA) equipped with a Thermo-scientific column (C18, 150 × 4.6 mm, 5 μm) and a photodiode array detector (Waters 2998, Milford, MA, USA) were used for analysis. The chromatograms were recorded and the astaxanthin peak appeared at 470 nm. Astaxanthin content was calculated from peak area using standards (25–100 ppm) and expressed as mg per 100 g oil.

### 2.8. Statistical Analysis

A completely randomized design was adopted for this study. All experiments were conducted in triplicate. Data analysis by ANOVA was performed using SPSS software (Statistical Package for Social Science, IBM software, New York, NY, USA). Duncan’s multiple range test was implemented for mean comparison.

## 3. Results and Discussion

### 3.1. Particle Size, Poly-Dispersity Index (PDI) and Zeta Potential

The particle sizes for KBPI-KC-SO microcapsules obtained from the spray-drying of ultrasonicated and microfluidized KBPI-KC-SO emulsions with various amounts of SO are tabulated in Table 1. Different sizes of microcapsules were observed as the amount of SO varied (*p* < 0.05). With a higher amount of oil, larger microcapsules were obtained. When emulsions contained a lower quantity of SO, the surface to volume ratio for the droplets was high, thus allowing wall material (protein + polysaccharide) to occupy the surface of the droplets effectively. As the amount of SO in the system increased, a higher amount of wall material was required to surround the SO. As a result, the size of the emulsion droplets could not be reduced when there was lower surface to volume ratio for the droplets. Moreover, US microcapsules were smaller compared to MF counterparts when the same amount of SO was used. The larger size of the latter could be explained by the phenomenon of “overprocessing”. Overprocessing is an intense homogenization process that can cause re-coalescence of the emulsion to form larger droplets [34]. Overprocessing is more pronounced for high-pressure microfluidization than ultrasonication. Sonication had little or no effect on the overprocessing of emulsions when compared to microfluidization [35]. The re-coalescence of droplets after microfluidization could also be attributed to the temperature rise during microfluidization. The temperature during ultrasonication was controlled by using an ice-bath, whereas microfluidization had no temperature control due to a difficulty in operation. An increase in emulsion temperature caused destabilization of emulsions by affecting interfacial adsorption of an emulsifier [36]. SO nanoliposomes prepared by ultrasonication were smaller in size compared to those prepared by microfluidization [6].

PDI values for the different KBPI-KC-SO microcapsules are shown in Table 1. The PDI values for the microcapsules ranged between 0.32 and 0.40, indicating a moderate size distribution for the powders. Size variation of spray-dried powder particles is a common phenomenon that occurs because of uneven drying rate, varying droplet size of emulsions, non-uniform atomization, different wall material composition and emulsion destabilization at the high temperatures used for drying [37,38,39]. A larger particle size for the microcapsules resulted in a higher corresponding PDI value for both US and MF microcapsules. Gulzar et al. [30] reported PDI values of 0.372 to 0.403 for spray-dried microcapsules loaded with mixed shrimp oil and tea seed oil where a higher PDI was related to larger microcapsules. PDI values of 0.468 and 0.705 corresponding to the particle size of 0.44 µm and 1.23 µm, respectively, were reported by Agustinisari et al. [40] for spray-dried whey protein-maltodextrin microcapsules loaded with eugenol.

The values for the ζ potential of the US and MF microcapsules are shown in Table 1. All microcapsule samples exhibited a negative charge on the surface, suggesting that high stability of the microcapsules was caused by electrostatic repulsion. The presence of negatively charged moieties on the microcapsules was attributed to the solubility of KBPI at pH 10. At a pH higher than the pI of KBPI, the proteins became deprotonated. The microcapsules became less negatively charged as the oil to wall material ratio decreased, implying the significance of the charged protein wall material for the ζ potential. ζ potential values for the microcapsules were also found to govern the encapsulation efficiency (EE) of the microcapsules (Table 1). Lower EE values indicated the presence of a high amount of surface oil on the microcapsules, which could substantially reduce the overall charge of the microcapsules by masking the surface negative charge. ζ potential values for spray-dried mung bean protein-sodium alginate microcapsules loaded with shrimp oil and tea seed oil were more negatively charged as the EE increased [30]. ζ potential values also showed that chitosan nanoparticles loaded with clove oil became less positively charged as the EE of the nanoparticles decreased [41].

### 3.2. Encapsulation Efficiency (EE)

The EE of the microcapsules ranged between 43.99% and 89.25% (Table 1). Higher EE of SO was found for microcapsules containing a higher wall material to oil ratio (less amount of SO). EE was directly influenced by the amount of protein-polysaccharide to oil content in emulsion. Numerous studies have shown that the amount and type of wall material play an important role in the EE of core materials. Gulzar et al. [30] reported higher EE of mixed tea seed oil and shrimp oil in mung bean protein and sodium alginate microcapsules when a higher ratio of protein-polysaccharide to oil was used. In another study, the EE of piperine in zein-xanthan gum microcapsules was augmented when an increased zein concentration was used [42]. The high solubility of protein in water also affects the EE by enhancing the emulsifying property of protein. As higher amounts of protein chains are diffused at the oil/water interface, the emulsion is stabilized in the form of small oil droplets [43]. KBPI is highly soluble in water at pH 10 (86.38–93.2%) [44]. Apart from the stabilization of emulsion by hydrophobic protein-protein interactions, the addition of κ-carrageenan forms additional protein-polysaccharide complexes via electrostatic interactions, which further enhanced the emulsification and stabilization of SO. Anionic polysaccharide-protein complexes induce cooperative adsorption at the oil interface, which further leads to the stabilization of emulsion. Furthermore, the anionic charge of polysaccharides inhibits aggregation of the droplets [25]. Polysaccharide incorporation also retards the coalescence of oil droplets by thickening the aqueous phase [24]. Irrespective of the wall material to oil ratio, the microcapsules prepared from ultrasonicated KBPI-KC-SO emulsions had higher EE (*p* < 0.05) compared to the microfluidized counterparts. These results were in agreement with the finding of Gulzar and Benjakul [6], who reported higher EE of nanoliposomes prepared by ultrasonication than those prepared by microfluidization. Ultrasonication resulted in a smaller particle size for the microcapsules than microfluidization (Table 1). Smaller microcapsules are indicative of smaller emulsion droplets corresponding to a larger surface to volume ratio and enhanced stability [15]. Anwar and Kunz [45] reported that increasing emulsion droplet size resulted in a higher amount of non-encapsulated oil content after spray-drying. 

### 3.3. Flowability

Table 2 depicts the Hausner ratio (HR) and flow behavior of KBPI-KC-SO microcapsules. Microcapsule samples were classified into three categories viz good flow, fair flow and passable flow, based on the HR values. US1 and MF1 were found to possess a good flow property, whereas US2 and MF2 had fair flow behavior. US3 and MF3 exhibited passable flow behavior. The good flow property of US1 and MF1 samples was attributed to the higher EE and less surface oil on the microcapsules. A higher amount of surface oil causes agglomeration of microcapsules and also lowers solubility and wettability [46]. Flowability, solubility and wettability of powders are among the important functional properties that determine the quality of food powders. HR is also an indicator of the friction condition between powders [47]. Food powders that are hygroscopic or contain surface oil experience low friction and therefore form lumps. The formation of lumps further hampers the reconstitution of powders in water [48]. Gulzar et al. [30] documented that mung bean protein-sodium alginate microcapsules loaded with a shrimp oil/tea seed oil mixture having a higher EE possessed better flowability, as characterized by low Carr index values. Spray-dried shrimp oil nanoliposome powder having a higher EE also showed a better flow property (low Carr index), high solubility and a lower reconstitution time [46]. Fitzpatrick et al. [49] demonstrated that spray-dried milk powder with a high surface fat content was more cohesive and formed agglomerates to a higher extent when compared to low-fat milk powders. The results for the KBPI-KC-SO microcapsule samples suggested acceptable flowability, particularly for US1 and MF1.

### 3.4. Microstructure

**Figure 1:**
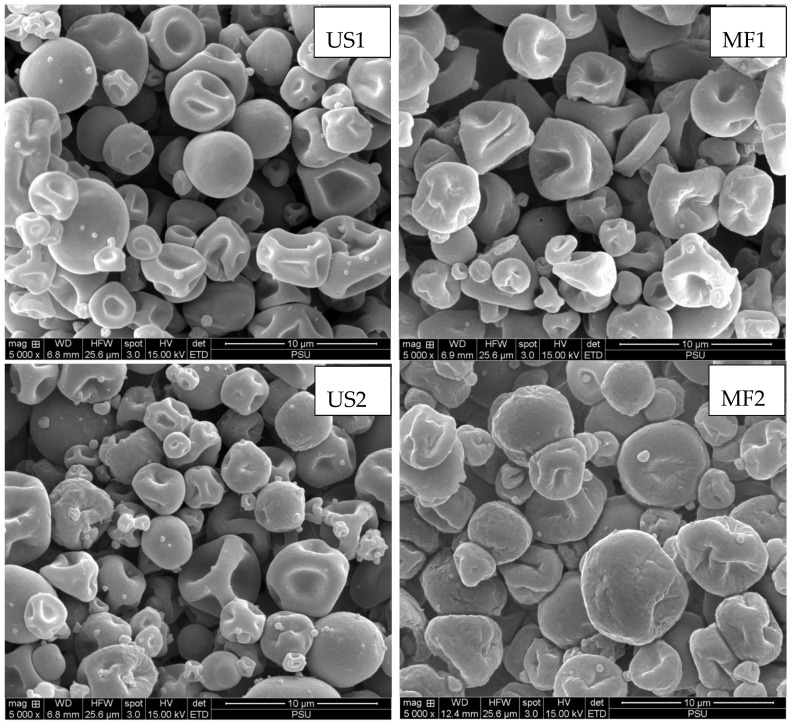

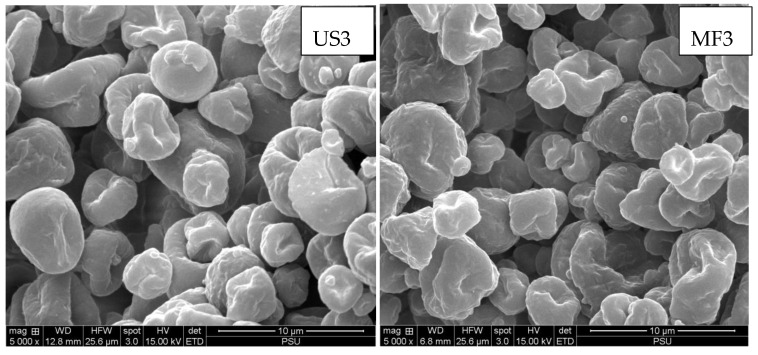
Scanning electron microscopic images of spray-dried microcapsules with KBPI and KC (1:0.1, *w*/*w*) as wall materials loaded with different amounts of SO. SO, shrimp oil, KBPI, kidney bean protein isolate; KC, κ-carrageenan; US1, ultrasonicated KBPI-KC-SO microcapsules containing 0.1% SO; US2, ultrasonicated KBPI-KC-SO microcapsules containing 0.5% SO; US3, ultrasonicated KBPI-KC-SO microcapsules containing 1% SO; MF1, microfluidized KBPI-KC-SO microcapsules containing 0.1% SO; MF2, microfluidized KBPI-KC-SO microcapsules containing 0.5% SO; MF3, microfluidized KBPI-KC-SO microcapsules containing 1% SO.

SEM images of US and MF KBPI-KC-SO microcapsules are illustrated in Figure 1. The microcapsules were generally spherical with surface wrinkles and indentations. The formation of surface indentations is characteristic of spray-dried powders caused by differential shrinkage at the surface and core of the microcapsules [50,51]. Uneven and rapid drying along with the nature of the wall materials affects the surface morphology of microcapsules [52]. It was also observed that the surfaces of some microcapsules were smoother than others, particularly US1 and MF1. US1 and MF1 had the highest EE and lowest surface oil. Therefore, these microcapsules had a smoother surface. As the surface oil on the microcapsules increased (with decreasing EE), the surface became irregular and wrinkled. The SEM images therefore visually substantiated the EE of the microcapsules. When comparing US and MF microcapsules, the former had slightly smoother surfaces. The overprocessing and rapid temperature rise in microfluidization could plausibly cause the denaturation of proteins, resulting in the destabilization of emulsions. Furthermore, the US1 and MF1 microcapsules appeared to show very little agglomeration compared to the other samples. This might be associated with less surface oil, thus preventing the agglomeration or attachment between microcapsules. This related well with the differences in flowability for the different microcapsules (Table 2).

For further characterization and storage studies, US1 and MF1 samples were selected based on their high EE and good flowability.

### 3.5. FTIR Spectra

**Figure 2:**
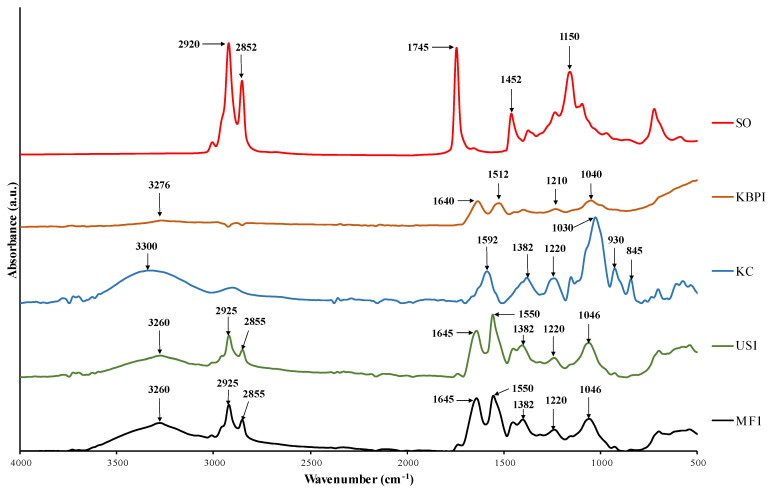
FTIR spectra of SO, KBPI, KC, US1 and MF1. SO, shrimp oil, KBPI, kidney bean protein isolate; KC, κ-carrageenan; US1, ultrasonicated KBPI-KC-SO microcapsules containing 0.1% SO; MF1, microfluidized KBPI-KC-SO microcapsules containing 0.1% SO.

FTIR spectra of US1 and MF1 samples in comparison with SO, KBPI and KC with characteristic peaks are illustrated in Figure 2. For SO, peaks at 2920 cm^−1^ and 2852 cm^−1^ correspond to the asymmetric stretching vibration of C–H of aliphatic CH_3_ and CH_2_ and symmetric C–H vibrations of aliphatic CH_2_ functional groups in triglycerides, respectively [53]. The stretching vibration peak at 1745 cm^−1^ is assigned to the esterified C=O bonds of fatty acid chains and the backbone of glycerol [53]. The peak at 1452 cm^−1^ represents the free fatty acids of SO, which is also used to determine the degree of hydrolysis in the oil [32]. The stretching vibration peak at 1150 cm^−1^ corresponds to the phosphate group of phospholipids that are abundantly present in shrimp oil [30]. The KBPI spectrum showed characteristic protein isolate bands corresponding to amide-A at 3276 cm^−1^. Two prominent peaks were seen at 1640 cm^−1^ and 1512 cm^−1^, which have been attributed to C=O stretching (amide-I region at 1640 cm^−1^) and N–H bending (amide-II region 1512 cm^−1^), respectively [54]. Additionally, some peaks were also observed at 1210 cm^−1^ and 1040 cm^−1^, which are likely due to the C–O stretching modes of ester bonds [55]. The FTIR spectra of KC showed several characteristic bands. Broad stretching bands at 3000-3500 cm^−1^ represent O–H stretching. KC showed prominent bands in the region of 1220–845 cm^−1^, known as the fingerprint region of carbohydrates, and had bands specific for each polysaccharide, which helps in their identification [56]. The peak at 1220 cm^−1^ corresponds to the asymmetric stretching of ester sulfate groups (O=S=O); 1030 cm^−1^ is assigned to C–O and C–OH stretching; 930 cm^−1^ corresponds to C–O–C stretching in 3,6 anhydro-d-galactose [57]. The band at 845 cm^−1^ is related to C–O–SO_3_ stretching in (1-3)-D-galactose [58]. The intense bands at 1592 cm^−1^ and 1382 cm^−1^ are plausibly related to the structural water deformation band [59].

For the US1 and MF1 spectra, all the major peaks for SO, KBPI and KC were detected in both samples. A small displacement or shift in the KBPI-associated bands in US1 and MF1 was observed for amide-A (from 3276 cm^−1^ to 3260 cm^−1^), amide-I (from 1640 cm^−1^ to 1645 cm^−1^) and amide-II (from 1512 cm^−1^ to 1550 cm^−1^), suggesting a chemical interaction between KBPI and KC or aggregation of KBPI. A shift in these bands has also been attributed to the unfolding of the protein structure, particularly during high-pressure microfluidization and the high temperature used for spray-drying. Ahmed et al. [54] reported that high-pressure treatment of KBPI resulted in a shift of the amide bands. Comparing the US1 and MF1 samples, a larger amplitude of bands corresponding to SO were observed in MF1, which could be related to the slightly higher amount of surface oil present in MF1. Overall, the FTIR results substantiated the presence of encapsulated SO in KBPI-KC microcapsules.

### 3.6. Oxidative Stability of SO 

#### 3.6.1. Lipid Oxidation

**Figure 3:**
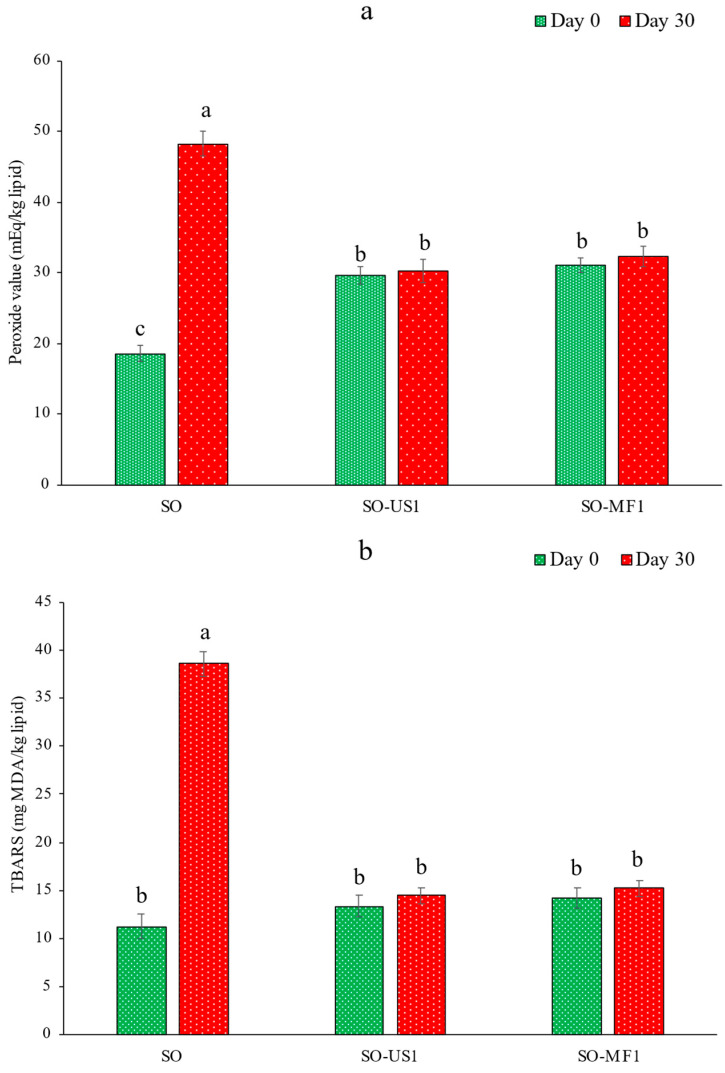
Peroxide (**a**) and TBARS values (**b**) for SO, SO-US1 and SO-MF1 at day 0 and day 30 of storage at room temperature (28–30 °C). Bars represent the standard deviation (*n* = 3). Different lowercase letters on the bars denote significant difference (*p* < 0.05). SO, shrimp oil; SO-US1, shrimp oil extracted from ultrasonicated KBPI-KC-SO microcapsules containing 0.1% SO; SO-MF1, shrimp oil extracted from microfluidized KBPI-KC-SO microcapsules containing 0.1% SO.

Figure 3a,b depict the peroxide value (PV) and TBARS value, respectively, for SO and the SO extracted from microcapsules including US1 (SO-US1) and MF1 (SO-MF1) at day 0 and day 30 of storage at room temperature. The PV of SO increased by over two-fold during 30 days of storage, whereas SO-US1 and SO-MF1 showed comparatively lower PV values (*p* < 0.05). The results suggested that encapsulation of SO in KBPI-KC microcapsules significantly prevented the oxidation of SO by forming a protective covering around the oil at the core. In general, marine oils are particularly rich in PUFAs and undergo rapid oxidation as reflected by an increase in PV [5,6,32,60]. Encapsulation of oil forms a barrier that prevents the exposure of oil to oxygen and other prooxidants [4,30,61,62]. An increase in the PV of SO-US1 and SO-MF1 was observed at day 0, which could be attributed to the deteriorative effect of high temperature exposure during spray-drying. However, no significant difference in the PV was observed between SO-US1 and SO-MF1 samples before and after storage (*p* > 0.05). A similar trend was observed in the TBARS values for the oil samples. The TBARS value of SO was augmented by three-fold after 30 days of storage. However, no change in the TBARS values for SO-US1 and SO-MF1 took place over the storage period (*p* > 0.05). The increased TBARS value for SO is indicative of the presence of carcinogenic secondary oxidation products (aldehydes and ketones) [63]. Overall, the results indicated the excellent protective property of encapsulation against SO deterioration induced by oxidation.

#### 3.6.2. Fatty Acid Profile

Table 3 shows the fatty acid profile for SO, SO-US1, and SO-MF1 on day 0 and day 30 of storage. Palmitic acid was the most abundant fatty acid in SO. SO contained high quantities of PUFAs, including EPA and DHA. Linoleic acid was also present in SO at a high level. At day 0, SO, SO-US1 and SO-MF1 had similar fatty acid profiles. However, there was a slight decrease in the PUFA content in SO-US1 and SO-MF1 at day 0. There was a concomitant marginal increase in PV in SO-US1 and SO-MF1 during spray-drying (Figure 3a). The fatty acid profile at day 30 showed a drastic decline in PUFA content in SO (*p* < 0.05). The unencapsulated SO underwent significant oxidation, resulting in the loss of PUFAs. On the other hand, SO-US1 and SO-MF1 retained PUFAs to a larger extent. At the end of storage period, there was 51.01% loss of PUFA from SO, whereas the PUFA content in SO-US1 and SO-MF1 decreased by 4.63% and 4.86%, respectively. Encapsulation was therefore proven to be an effective technique for retaining valuable PUFAs in SO. Encapsulation of marine oils prevents substantial loss of PUFAs and enhances the quality of several oils [6,22,46,61,64]. The loss of PUFAs from SO also resulted in an increase in the saturated fatty acid (SFA) content of the oil. It was reported by Gulzar and Benjakul [32] that oxidation of PUFAs caused a subsequent increase in the SFA content of SO. MUFAs of SO were markedly decreased after storage for 30 days. Only a slight decrease in MUFAs was found for both SO-US1 and SO-MF1 on day 30. This reconfirmed that unsaturated fatty acids underwent less oxidation when encapsulated by wall materials.

#### 3.6.3. Astaxanthin

**Figure 4:**
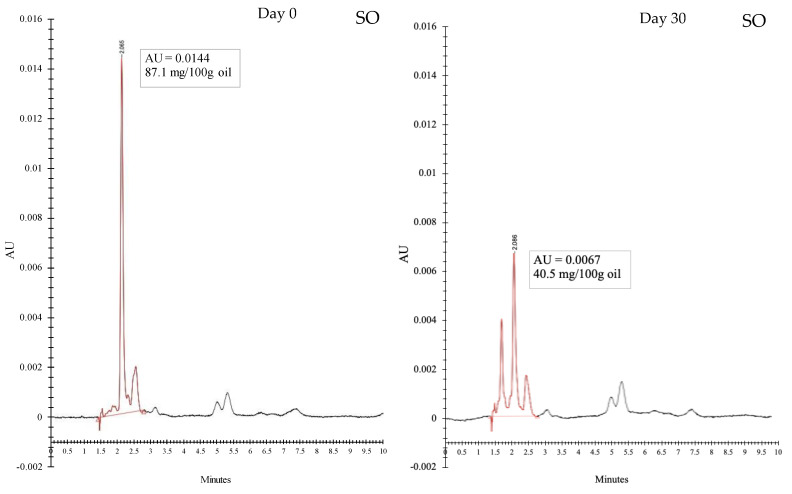

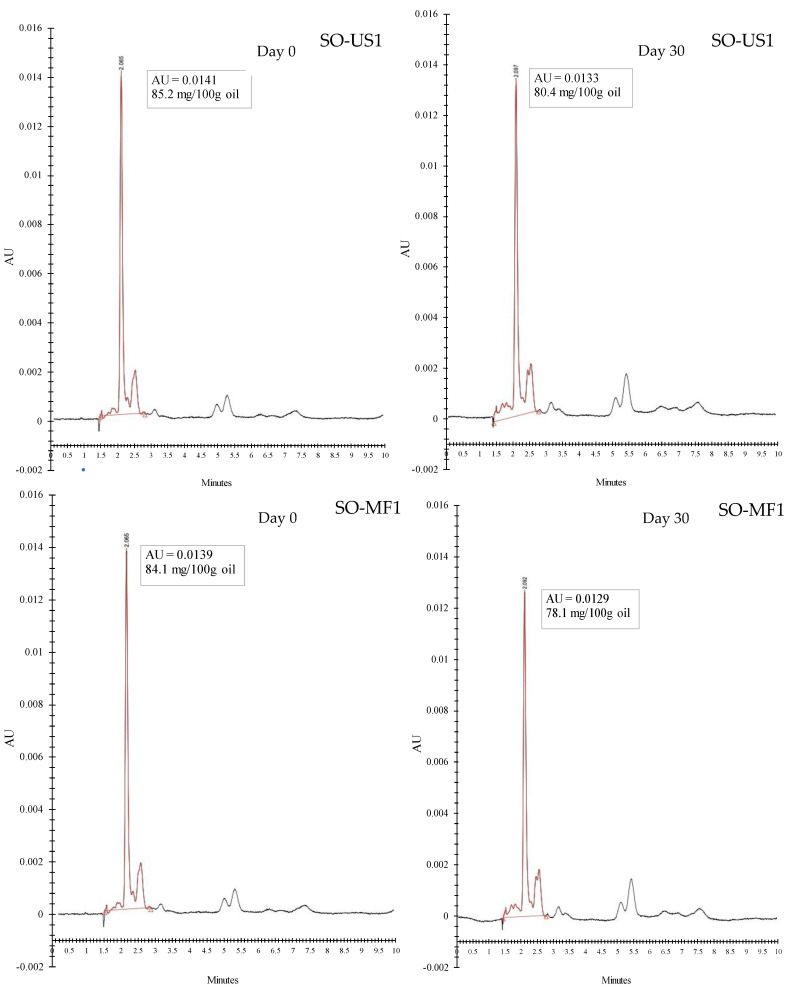
HPLC chromatograms of SO, SO-US1 and SO-MF1 at day 0 and day 30 of storage at room temperature (28–30 °C). SO, shrimp oil; SO-US1, shrimp oil extracted from ultrasonicated KBPI-KC-SO microcapsules containing 0.1% SO; SO-MF1, shrimp oil extracted from microfluidized KBPI-KC-SO microcapsules containing 0.1% SO. Astaxanthin content was calculated from peak area.

**Figure 5:**
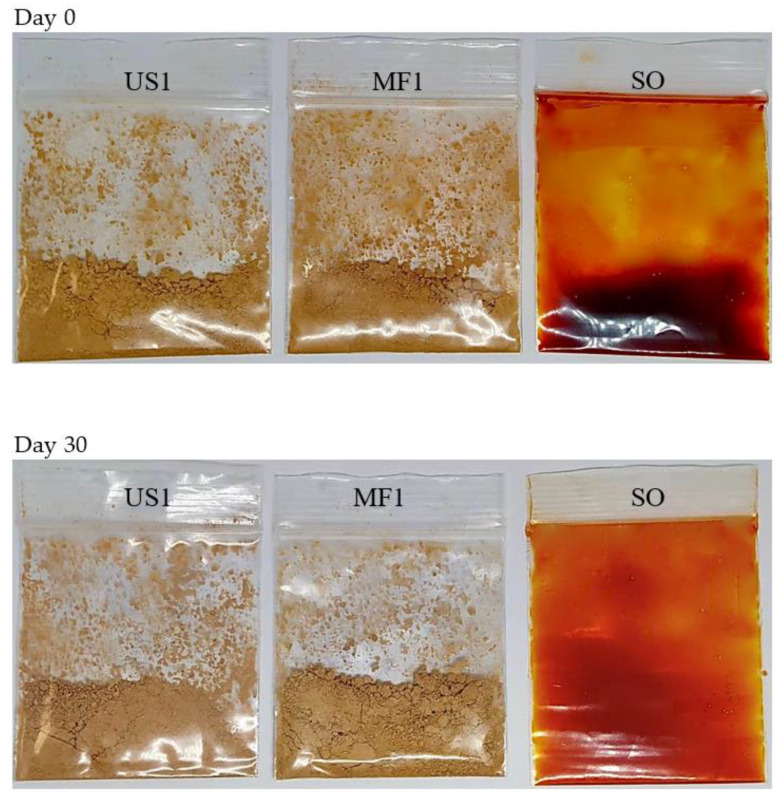
Images of SO, US1 and MF1 samples packed in zip lock bags at day 0 and day 30 of storage at room temperature (28–30 °C). SO, shrimp oil; US1, ultrasonicated KBPI-KC-SO microcapsules containing 0.1% SO; MF1, microfluidized KBPI-KC-SO microcapsules containing 0.1% SO.

HPLC chromatograms of SO, SO-US1 and SO-MF1 at day 0 and day 30 are illustrated in Figure 4. After 30 days of storage, the astaxanthin content of SO was reduced by more than 50%, indicating that decomposition of astaxanthin took place by oxidation. However, there was very little reduction in the astaxanthin content of SO-US1 and SO-MF1 after 30 days of storage. Encapsulation of SO in KPBI-SO microcapsules significantly lowered the oxidation of astaxanthin. Images of the storage bags containing SO, US1 and MF1 samples at day 0 and day 30 (Figure 5) also demonstrate the loss of redness from the SO sample caused by the decomposition of astaxanthin. The color of the SO sample at day 0 was dark reddish, however it turned into a much lighter in color by day 30. Astaxanthin is a highly potent antioxidant that is readily oxidized if not stored properly. Astaxanthin also plays a vital role in retarding lipid peroxidation and protects the PUFAs from oxidation [65]. Nevertheless, the astaxanthin content in the SO-US1 and SO-MF1 samples showed a slight decrease by day 30, more likely due to the oxidation of astaxanthin by surface oil present on the microcapsules. No obvious change in the color of the microcapsules in both US1 and MF1 was observed before and after storage (Figure 5). These results are consistent with the findings of Gulzar and Benjakul [6] who documented that the encapsulation of shrimp oil in nanoliposomes prevented the loss of astaxanthin content compared to free oil. Encapsulation of shrimp oil in chitosan-tripolyphosphate nanoparticles also helped to retain astaxanthin content during prolonged storage [64]. 

## 4. Conclusions

Emulsions of KBPI-KC-SO with varying amounts of SO were prepared by ultrasonication and high-pressure microfluidization, and subsequently spray-dried to entrap SO in KBPI-KC microcapsules. High encapsulation efficiency of KBPI-KC-SO microcapsules was observed, especially when a high protein-polysaccharide to oil ratio was used. Spherical microcapsules having some shrinkage with good flow behavior and high zeta potential were obtained. Encapsulation of SO in KBPI-KC microcapsules could retard lipid oxidation and retained PUFAs and astaxanthin in SO during extended storage for 30 days. Therefore, utilization of low-cost plant-based proteins along with the naturally abundant hydrocolloid κ-carrageenan as wall materials and microencapsulation is an effective means to protect valuable health promoting functional foods like shrimp oil from deterioration by the oxidation process.

## Figures and Tables

**Table 1:** Particle size, polydispersity index (PDI), zeta potential and encapsulation efficiency (EE) of spray-dried microcapsules having KBPI and KC (1:0.1, *w*/*w*) as wall materials loaded with different amounts of SO.

Microcapsule Samples	Wall Materials (*w*/*w*/*w*)	Size (µm)	PDI	Zeta Potential (mV)	EE (%)
US1	KBPI:KC:SO (1:0.1:0.1)	2.58 ± 0.26d	0.320 ± 0.03b	−58.77 ± 1.86a	89.25 ± 1.32a
US2	KBPI:KC:SO (1:0.1:0.5)	4.32 ± 0.44bc	0.389 ± 0.04a	−48.25 ± 2.06b	64.72 ± 1.38c
US3	KBPI:KC:SO (1:0.1:1)	4.24 ± 0.54b	0.327 ± 0.03a	−43.72 ± 1.43c	52.61 ± 2.01e
MF1	KBPI:KC:SO (1:0.1:0.1)	3.21 ± 0.25c	0.335 ± 0.04a	−49.59 ± 0.83b	86.42 ± 1.44b
MF2	KBPI:KC:SO (1:0.1:0.5)	4.91 ± 0.12b	0.384 ± 0.07a	−46.66 ± 0.70c	59.33 ± 1.27d
MF3	KBPI:KC:SO (1:0.1:1)	6.41 ± 0.81a	0.400 ± 0.05a	−35.95 ± 0.93d	43.99 ± 1.05f

Note: KBPI, kidney bean protein isolate; KC, κ-carrageenan; SO, shrimp oil; US1, ultrasonicated KBPI-KC-SO microcapsules containing 0.1% SO; US2, ultrasonicated KBPI-KC-SO microcapsules containing 0.5% SO; US3, ultrasonicated KBPI-KC-SO microcapsules containing 1% SO; MF1, microfluidized KBPI-KC-SO microcapsules containing 0.1% SO; MF2, microfluidized KBPI-KC-SO microcapsules containing 0.5% SO; MF3, microfluidized KBPI-KC-SO microcapsules containing 1% SO. Data are presented as mean ± SD (*n* = 3). Different lowercase letters in the same column indicate significant difference (*p* < 0.05).

**Table 2:** Flow behavior of spray-dried microcapsules having KBPI and KC (1:0.1, *w*/*w*) as wall materials loaded with different amounts of SO.

Microcapsule Samples	Hausner Ratio	Flow Behavior
US1	1.14 ± 0.03c	Good flow
US2	1.18 ± 0.02b	Fair flow
US3	1.28 ± 0.03a	Passable flow
MF1	1.13 ± 0.03c	Good flow
MF2	1.22 ± 0.02b	Fair flow
MF3	1.29 ± 0.03a	Passable flow

Note: KBPI, kidney bean protein isolate; KC, κ-carrageenan; SO, shrimp oil; US1, ultrasonicated KBPI-KC-SO microcapsules containing 0.1% SO; US2, ultrasonicated KBPI-KC-SO microcapsules containing 0.5% SO; US3, ultrasonicated KBPI-KC-SO microcapsules containing 1% SO; MF1, microfluidized KBPI-KC-SO microcapsules containing 0.1% SO; MF2, microfluidized KBPI-KC-SO microcapsules containing 0.5% SO; MF3, microfluidized KBPI-KC-SO microcapsules containing 1% SO. Data are presented as mean ± SD (*n* = 3). Different lowercase letters in the same column indicate significant difference (*p* < 0.05).

**Table 3:** Fatty acid profile of SO and SO extracted from US1 and MF1 on day 0 and day 30 of storage at room temperature (28–30 °C).

Fatty Acids (%)	Day			Day 30		
	SO	SO-US1	SO-MF1	SO	SO-US1	SO-MF1
C14:0 (Myristic)	1.11 ± 0.06b *	1.09 ± 0.03b	1.10 ± 0.02b	1.32 ± 0.06a	1.13 ± 0.03b	1.14 ± 0.07b
C15:0 (Pentadecanoic)	0.23 ± 0.01c	0.30 ± 0.04b	0.29 ± 0.02b	0.49 ± 0.05a	0.32 ± 0.04b	0.31 ± 0.04b
C16:0 (Palmitic)	10.98 ± 0.15b	11.06 ± 0.19b	11.11 ± 0.13b	14.36 ± 0.09a	11.21 ± 0.11b	11.19 ± 0.15b
C16:1 (Palmitoleic)	0.83 ± 0.02a	0.91 ± 0.07a	0.93 ± 0.09a	0.73 ± 0.05b	0.82 ± 0.11a	0.81 ± 0.09a
C17:0 (Heptadecanoic)	0.81 ± 0.03c	0.84 ± 0.03bc	0.85 ± 0.04bc	1.36 ± 0.10a	0.96 ± 0.04b	0.98 ± 0.03b
C17:1 cis 10 (cis-10-Heptadecanoic)	0.22 ± 0.02a	0.23 ± 0.02a	0.22 ± 0.01a	0.18 ± 0.01b	0.21 ± 0.01ab	0.20 ± 0.02ab
C18:0 (Stearic)	3.02 ± 0.07b	3.09 ± 0.10b	3.15 ± 0.13b	3.63 ± 0.17a	3.25 ± 0.12b	3.21 ± 0.09b
C18:1 (Oleic)	4.28 ± 0.13b	4.26 ± 0.10b	4.19 ± 0.11b	3.73 ± 0.16a	4.23 ± 0.08b	4.18 ± 0.15b
C18:2 (Linoleic)	5.76 ± 0.21a	5.69 ± 0.18a	5.77 ± 0.12a	4.13 ± 0.11b	5.56 ± 0.13a	5.59 ± 0.09a
C18:3 (alpha-Linoleic)	0.67 ± 0.03a	0.69 ± 0.02a	0.68 ± 0.03a	0.39 ± 0.01b	0.66 ± 0.04a	0.67 ± 0.04a
C20:0 (Arachidic)	1.09 ± 0.04a	1.13 ± 0.02a	1.08 ± 0.03a	1.03 ± 0.01b	1.04 ± 0.03ab	1.05 ± 0.04ab
C20:1 (Eicosenoic)	0.72 ± 0.02a	0.69 ± 0.01a	0.70 ± 0.02a	0.59 ± 0.03b	0.66 ± 0.01a	0.67 ± 0.03a
C20:2 (Eicosadienoic)	1.04 ± 0.07a	1.01 ± 0.04a	0.99 ± 0.02a	0.39 ± 0.05b	0.97 ± 0.05a	0.95 ± 0.07a
C20:5 (EPA)	5.08 ± 0.15a	4.89 ± 0.10a	4.81 ± 0.14a	1.36 ± 0.11c	4.61 ± 0.08b	4.55 ± 0.14b
C22:6 n-3 (DHA)	6.58 ± 0.18a	6.49 ± 0.11a	6.43 ± 0.11a	3.19 ± 0.20c	6.14 ± 0.09b	5.99 ± 0.16b
C23:0 (Tricosanoic)	1.98 ± 0.13a	1.91 ± 0.07a	1.93 ± 0.06a	1.87 ± 0.14a	1.84 ± 0.06a	1.89 ± 0.06a
C24:1 (Nervonic)	0.56 ± 0.02a	0.55 ± 0.01a	0.53 ± 0.02a	0.29 ± 0.01c	0.44 ± 0.03b	0.46 ± 0.02b
Saturated fatty acid (SFA)	19.31 ± 0.17c	19.45 ± 0.19c	19.49 ± 0.13c	24.08 ± 0.19a	19.76 ± 0.17b	19.78 ± 0.19b
Monounsaturated fatty acid (MUFA)	6.67 ± 0.23a	6.64 ± 0.13a	6.58 ± 0.12a	5.55± 0.16c	6.31 ± 0.14b	6.33 ± 0.11b
Polyunsaturated fatty acid (PUFA)	19.29 ± 0.22a	18.78 ± 0.16b	18.70 ± 0.19b	9.45 ± 0.21d	17.91 ± 0.15c	17.79 ± 0.13c

Note: SO, shrimp oil; SO-US1, shrimp oil extracted from ultrasonicated KBPI-KC-SO microcapsules containing 0.1% SO; SO-MF1, shrimp oil extracted from microfluidized KBPI-KC-SO microcapsules containing 0.1% SO. Data are presented as mean ± SD (*n* = 3). * Different lowercase letters in the same row indicate significant difference (*p* < 0.05).

## Data Availability

The data presented in this study are available in article.
